# Therapeutic Potential of Proanthocyanidins in Dentistry: A Focus on Periodontal Disease and on Dental Implants in Osteoporotic Patients

**DOI:** 10.3390/antiox14070850

**Published:** 2025-07-10

**Authors:** Yoshimi Niwano, Shunichi Shishido, Midori Shirato, Hidetsugu Kohzaki, Keisuke Nakamura

**Affiliations:** 1 Faculty of Nursing, Shumei University, 1-1 Daigaku-cho, Yachiyo 276-0003, Chiba, Japan; kohzaki@mailg.shumei-u.ac.jp; 2 Department of Advanced Free Radical Science, Tohoku University Graduate School of Dentistry, 4-1 Seiryo-machi, Aoba-ku, Sendai 980-8575, Miyagi, Japan; shunichi.shishido.b2@tohoku.ac.jp (S.S.); midori.shirato.c8@tohoku.ac.jp (M.S.); keisuke.nakamura.e5@tohoku.ac.jp (K.N.)

**Keywords:** proanthocyanidins, oral health problems, osteoporosis, gut barrier function, Nrf2 signaling pathway

## Abstract

Proanthocyanidins (PACs), also called condensed tannins, are oligomers or polymers composed of flavan-3-ols. This review aimed to explore the potential role of PACs in ameliorating oral health problems, with a particular focus on their effects within the intestine—especially the colon, where most orally ingested PACs are believed to accumulate. Previous studies suggest that PACs can be beneficial in periodontal disease as well as in the osseointegration of dental implants in patients with osteoporosis. Periodontal disease is worsened by lipopolysaccharides (LPS) that enter the bloodstream due to disrupted tight junctions of intestinal epithelial cells, along with inflammatory cytokines released by activated macrophages. A similar mechanism is thought to affect osseointegration: LPS-induced inflammatory cytokines originating in the intestine can enter the bloodstream, contributing to bone loss and impaired integration of dental implants. PACs absorbed by intestinal epithelial cells can function as prooxidants, triggering the nuclear factor erythroid 2-related factor 2 (Nrf2) signaling pathway, which strengthens the gut barrier. This enhanced barrier reduces the levels of LPS and inflammatory cytokines in the blood, leading to the alleviation of periodontal inflammation and increased alveolar bone density, thereby promoting better osseointegration of dental implants.

## 1. Introduction

Proanthocyanidins (PACs), also called condensed tannins, are compounds that break down into red anthocyanidin pigments in acidic conditions. They are oligomers or polymers composed of flavan-3-ols, such as epicatechin and catechin. These substances are widely found in fruits, grains, and leaves, particularly in cocoa, black soybeans, cinnamon, apples, and grape seeds [1,2,3,4,5,6]. The average degree of polymerization of PACs from grape seeds ranges from 2 to 17 [1]. As observed in our previous review, when oligomeric and polymeric PACs are consumed orally, a significant portion reaches the colon, where a small fraction undergoes microbial degradation into phenolic acids and valerolactones [7]. This occurs despite the possibility of slight depolymerization of PACs in the stomach and small intestine. In this narrative review, we aimed to explore whether MafPACs would improve oral health problems through the gut–oral axis. We conducted a comprehensive literature search across multiple scientific databases, including PubMed, Embase, and Web of Science. The search strategy incorporated a range of relevant keywords such as “proanthocyanidins,” “osteoporosis,” “oral health,” “dental implant,” “gut barrier function,” “gut microbiota,” and “Nrf2 signaling pathway,” among others. These keywords were selected to capture a broad range of studies spanning basic, translational, and clinical research related to the biological functions of PACs and their potential systemic and oral health implications.

## 2. Effect of Proanthocyanidins on Gut Barrier Function

Considering the metabolic fate of orally ingested PACs, the colon has been considered the major biological target [7]. From this point of view, several in vitro studies have been conducted using colon cells. An in vitro study using human colon cell lines (Caco-2 and HT-29 cells) showed that PACs, especially higher-molecular-weight polymers, were effective in maintaining membrane integrity and suppressing interleukin (IL)-8 secretion in response to inflammatory stimuli [8]. Another in vitro study showed that a PAC-rich grape seed extract (GSE) enhanced the expression of tight junction proteins in LPS-treated Caco-2 cells, promoted the expression of anti-inflammatory cytokines, and suppressed the gene expression of pro-inflammatory cytokines [9,10]. These findings suggest that PACs (especially polymeric ones) are effective in preventing the deterioration of gut barrier function and epithelial inflammation, both of which play a crucial role in the development of metabolic endotoxemia, inflammatory bowel disease, and colon cancer. Indeed, an in vivo study using obese rats induced by a high-fat/high-carbohydrate diet showed that orally administered GSE improved gut barrier dysfunction, ameliorating metabolic endotoxemia [11]. Similarly, oral GSE protected against dextran sulfate sodium (DSS)-induced colitis by preventing gut barrier dysfunction, as indicated by upregulated mRNA levels encoding tight junction proteins including zonula occludens protein 1 (ZO-1), occludin, and claudin-1 of colon tissue, in addition to reduced oxidative stress and inflammatory cytokines [12]. Several in vivo studies have also demonstrated that PACs ameliorate gastrointestinal and metabolic diseases with improved gut barrier functions [12,13,14,15].

**Figure 1:**
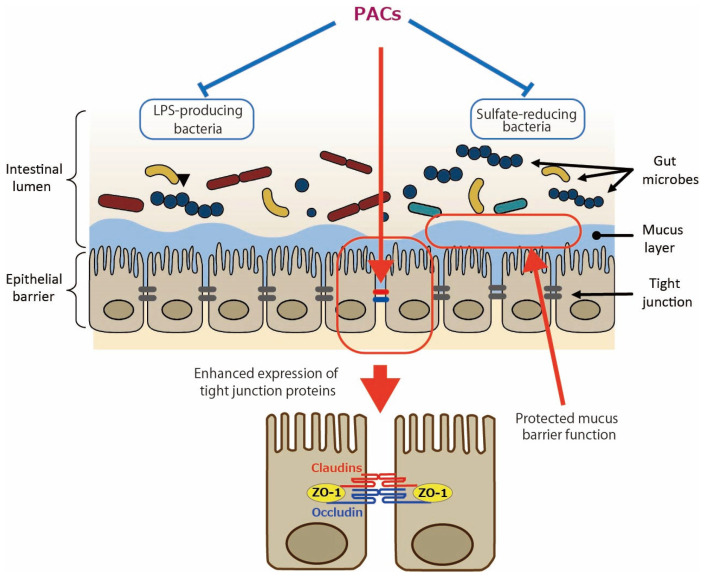
Enhanced gut barrier function by proanthocyanidins (PACs). PACs could improve mucus barrier function by modulating gut microbiota such as reducing sulfate-reducing bacteria. PACs could also directly upregulate the expression of tight junction proteins such as claudins, occludin, and zonula occludens protein 1 (ZO-1). Blue lines indicate inhibitory sites, and red lines indicate sites of action.

In addition to the direct effect of PACs on intestinal epithelial cells as shown by the aforementioned in vitro studies, the involvement of gut microbiota in the effects of PACs has been studied. In the in vivo study of DSS-induced colitis [12], an analysis of 16S rRNA sequences showed that GSE rebalanced the DSS-damaged gut microbiota: at the phylum level, it reduced Bacteriodota (formerly named Bacteroidetes) with the elevated Bacillota (formerly named Firmicutes)/Bacteroidota ratio; at the genus level, it reduced *Dubosiella* and *Veillonella* and increased *Verrucomicrobia* and *Akkermansia*. In type 2 diabetes mellitus (T2DM) mice, PACs enhanced the relative abundance of Lachnospiraceae at the family level; *Lachnospiraceae_NK4A136_group*, *Alloprevotella*, *Akkermansia*, and *Faecalibaculum* at the genus level; and reduced that of Bacteroidota and Muribaculaceae at the phylum and family levels, respectively [14]. Regarding the Bacteroidota phylum, since it is the main Gram-negative bacteria that can release endotoxins leading to metabolic endotoxemia [16], the authors suggested that a reduced amount of Bacteriodota may lower serum LPS levels. The Lachnospiraceae family and *Lachnospiraceae_NK4A136_group* genus, both types of butyrate-producing bacteria, have been shown to support gut barrier integrity in mice and are inversely related to intestinal permeability [17]. The *Alloprevotella* genus generates acetate and butyrate, which are negatively correlated with metabolic syndromes like obesity, diabetes, and cardiovascular diseases in humans [18]. In high-fat diet (HFD)-induced obese mice, it was shown that PACs decreased the percentage of an endotoxin-producing and sulfate-reducing bacterial family, Desulfovibrionaceae [15]. Hydrogen sulfide (H_2_S) produced by the Desulfovibrionaceae family may reduce disulfide bonds in the intestinal mucus layer, compromising the mucus barrier and exposing the epithelium to bacteria and toxins, which could potentially lead to inflammation [19]. The proposed mechanisms by which PACs improve gut barrier function are illustrated in Figure 1.

In terms of interactions with the gut microbiota, the following points must be taken into consideration. Although oral administration of PACs is commonly reported, they also have well-documented pharmacokinetic limitations. Oligomeric PACs generally exhibit poor intestinal absorption and are subject to extensive microbial metabolism in the gut, leading to the production of lower-molecular-weight metabolites [7]. In in vitro fermentation studies using PAC-rich GSEs, the highest levels of intermediate metabolites—such as valerolactones, valeric acid, various phenolic acids, and gallic acid—were observed after 5–10 h of incubation with fecal microbiota. During the later incubation period (10–48 h), mono- and non-hydroxylated derivatives of these metabolites began to appear [20,21]. These in vitro findings are consistent with those from a human study, in which participants consumed a test drink containing PACs with degrees of polymerization (DP) ranging from 2 to 10; in this study, γ-valerolactones were the main metabolites detected in plasma [22]. These metabolites may contribute to systemic biological effects, leading to the possibility that the therapeutic impact of PACs in periodontal disease may depend to some extent on the activity of these microbial-derived metabolites in addition to the parent compounds themselves. Regarding the distinction between polymeric and dimeric PACs, another human study investigated the metabolic fate of (−)-epicatechin, PAC B1 (a dimer), and polymeric PACs, all encapsulated in hard gelatin to minimize oral and gastric interactions [23]. After ingestion of PAC B1, free PAC B1, 4-hydroxy-5-(3′,4′-dihydroxyphenyl)valeric acid, and 5-(3′,4′-dihydroxyphenyl)valerolactone were detected in plasma. In contrast, no dimeric or oligomeric PACs were found in plasma following ingestion of polymeric PACs (mean degree of polymerization: 5.9). This indicates that 5-(3′,4′-dihydroxyphenyl)valerolactone is a major microbial metabolite of PAC B1. A small portion of PAC B1 also underwent phase II metabolism after entering circulation. The large molecular size of polymeric PACs likely limits microbial degradation owing to poor bacterial uptake. These findings showed that polymeric PACs remained intact in the colon and exerted beneficial local effects.

## 3. Amelioration of Periodontal Diseases by Proanthocyanidins Through Gut Barrier Function

Recent epidemiological studies suggest that periodontal disease is a risk factor for systemic diseases such as diabetes, atherosclerotic diseases, and rheumatoid arthritis [24,25,26,27,28,29,30,31,32,33,34,35,36,37]. The common mechanism is the enhancement of systemic inflammatory responses via the transfer of LPS and inflammatory cytokines derived from periodontal disease-causing bacteria such as *Porphyromonas gingivalis*; however, sufficient evidence has not yet been obtained. The intestinal microbiota are thought to be 600 to 1000 trillion in number, and there are >1000 species of commensal bacteria living in the human intestine. It has been suggested that the intestinal microbiota are involved in various metabolic processes in the host, influencing metabolic pathologies such as obesity and metabolic syndrome, as well as the development and function of the nervous system [38,39,40,41,42,43]. The mechanism by which dysbiosis of the intestinal microbiota exacerbates T2DM is speculated to be partly due to the induction of inflammation via endotoxemia, which is caused by the transfer of LPS into the blood due to impaired intestinal barrier function [44,45,46]. Regarding dysbiosis and atherosclerosis, a scenario where atherosclerosis is exacerbated through multiple pathways, including short-chain fatty acids, LPS, and trimethylamine-N-oxide produced by intestinal bacteria, has been postulated [47,48,49,50]. Although it has been speculated that intestinal dysbiosis and impaired intestinal barrier function may aggravate rheumatoid arthritis, this has not yet been proven [51,52].

**Figure 2:**
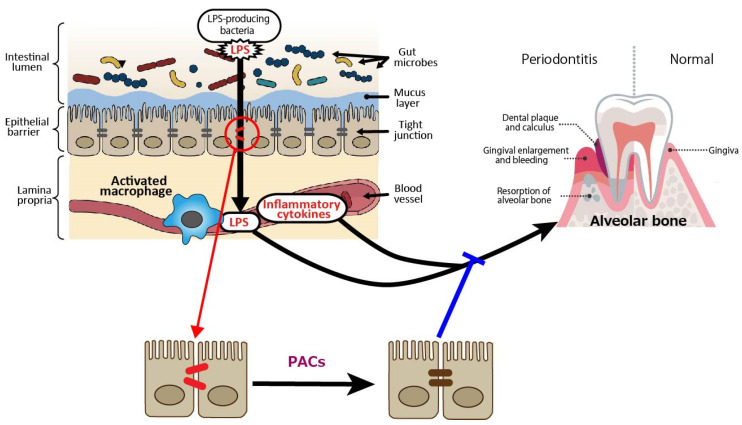
Intestine-derived lipopolysaccharide (LPS) and inflammatory cytokines as aggravating factors in periodontal diseases, and preventive effect of proanthocyanidins (PACs) on periodontal diseases. LPS leaks into the blood from the site of tight junction disruption, and inflammatory cytokines are produced by activated macrophages, which may affect periodontal tissue. PACs enhance epithelial barrier functions, suppressing disease exacerbation by blocking LPS leakage. A blue line indicates an inhibitory site, and a red line indicates a site of action.

In terms of periodontal disease from the gut bacteria perspective, it has been reported that DSS-induced enteritis in mice, a common model of human inflammatory bowel disease that exhibits macrophage activation in the colon, impaired intestinal barrier function, and increased Gram-negative bacteria [53,54], with increased alveolar bone resorption over time [55]. Macrophage activation, reduced intestinal barrier function, and increased Gram-negative bacteria indicate that LPS leaks into the blood from the site of tight junction disruption, and inflammatory cytokines are produced by activated macrophages, which may also affect periodontal tissue. PACs are expected to prevent the exacerbation of periodontal diseases by improving gut barrier function (Figure 2).

## 4. Enhancement of Osseointegration of Dental Implants in Patients with Osteoporosis by Proanthocyanidins Through Gut Barrier Function

Osteoporosis is a systemic metabolic bone disease characterized by a reduction in bone mass and the deterioration of bone microstructure, which increases bone fragility and the risk of fractures. Postmenopausal osteoporosis (PMOP) accounts for a significant proportion of cases. Numerous studies have shown that estrogen deficiency is associated with an imbalance in gut microbiota, a compromised intestinal mucosal barrier, and heightened inflammatory responses, and, as such, estrogen therapy may help lower the risk of PMOP by preventing increased permeability of the gut epithelium, bacterial translocation, and age-related inflammation [56]. In an animal study, fecal microbiota transplantation (FMT) from a healthy donor to ovariectomized (OVX) mice as a PMOP model suppressed excessive osteoclastogenesis and protected against OVX-induced bone loss. Specifically, compared to the OVX group, FMT increased the expression of tight junction proteins (ZO-1 and occludin) while reducing the secretion of inflammatory cytokines, including tumor necrosis factor-α (TNF-α) and IL-1β [57]. Similarly, it was shown that OVX-induced estrogen deficiency in mice significantly altered the composition of gut microbiota, resulting in severe impairment of intestinal barrier function and increased gut permeability [58]. Consequently, LPS-induced inflammatory cytokines released from the intestine into the bone marrow were found to be linked to bone loss in OVX mice [59]. Receptor activator of nuclear factor kappa B (NF-κB) ligand (RANKL), which is secreted by osteoblasts and plays a crucial role in osteoclast development and activation, binds to its receptor RANK, triggering NF-κB activation and promoting osteoclast differentiation at an accelerated rate [60,61,62]. Inflammatory cytokines such as IL-1β, and TNF-α have been proposed as key contributors to the accelerated bone loss observed during menopause. The increased production of these cytokines is linked to the interaction between RANKL and RANK, which triggers osteoclastic bone resorption in the absence of estrogen [63].

Based on this background, we discuss the relationship between osteoporosis and dental implants. Some studies have shown that osteoporosis is a risk factor for the success of dental implantation surgery [64,65,66,67,68]. A prospective, controlled, multicenter study showed that implants placed in patients with osteoporosis had a significantly higher failure rate over a 5-year follow-up period compared to non-osteoporotic controls on an implant level but not on a patient level [69]. More recently, a systematic review with meta-analysis concluded that dental implants serve as an effective treatment option for rehabilitating patients with osteoporosis; however, professional clinical care is essential to maintaining peri-implant bone stability, as these patients may have a higher risk of bone loss [70]. Another recent review article focusing on jaw osteoporosis [71,72]—which can result from microstructural damage to the alveolar bone owing to systemic osteoporosis—revealed that jaw osteoporosis reduces implant survival rates, compromises initial implant stability, and increases peri-implant bone loss [73].

**Figure 3:**
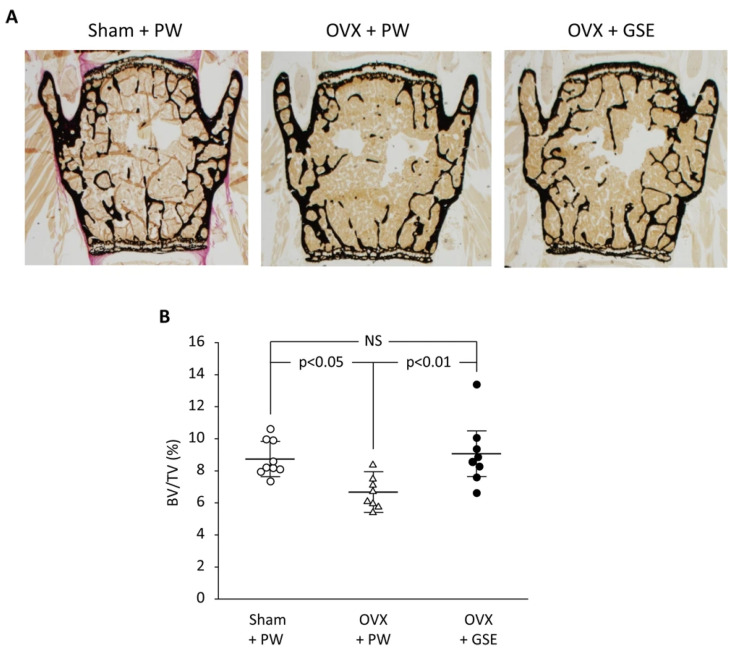
Effect of oral administration of proanthocyanidin-rich grape seed extract (GSE) on bone loss in the third and fourth lumbar vertebrae from spines of ovariectomized (OVX) mice. (**A**) Representative images of von Kossa-stained third vertebrae from each of the three groups. (**B**) The trabecular bone volume/total volume (BV/TV) ratio was determined by histological analysis of bones in the lumbar spine. In (**B**), the results are expressed as mean ± standard deviation, showing individual data (n = 9 in Sham + PW, n = 8 in OVX + PW and OVX + GSE). PW: pure water, NS: not significant (*p* > 0.05). Adapted from Tenkumo et al. [75], Scientific Reports, (2020), doi.org/10.1038/s41598-020-65403-4, used under CC BY 4.0.

**Figure 4:**
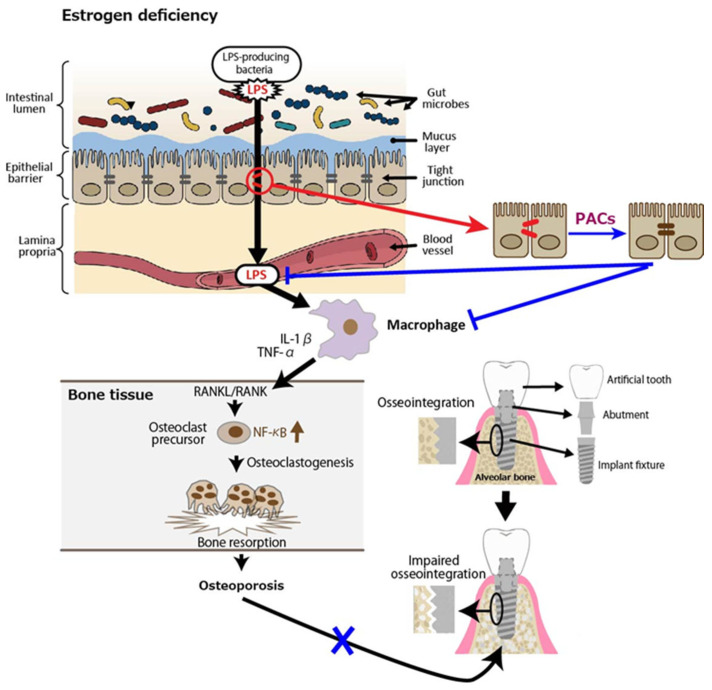
Postmenopausal osteoporosis as a putative risk factor for impaired osseointegration leads to lower success rates of dental implants. Estrogen deficiency reduces intestinal barrier function, leading to lipopolysaccharides (LPS) in the blood. LPS activates macrophages to secrete inflammatory cytokines such as interleukin (IL)-1β and tumor necrosis factor (TNF)-α, which interact with receptor activator of nuclear factor kappa beta (NF-κB) ligand (RANKL)/RANK, triggering NF-κB activation and osteoclastogenesis. The resultant osteoporosis could impair osseointegration of the alveolar bone and dental implants. Blue lines indicate inhibitory sites.

Finally, we discuss the possible involvement of PACs in improving osseointegration of dental implants in patients with osteoporosis. PACs are well known for their antioxidant effects, which mainly function by reducing oxidative stress through the autoxidation of their phenolic hydroxyl groups. PMOP, caused by estrogen deficiency, is closely linked to oxidative stress. Therefore, PACs may offer a safe and effective treatment option. As stated in our previous review, polyphenols including PACs show anti-inflammatory and antioxidant effects, which block the RANKL/RANK interaction through the NF-κB pathway, reducing bone resorption [74]. We also showed using OVX mice and rats that GSE can exert a beneficial effect on bone health [75]. That is, daily oral supplementation with GSE was shown to prevent bone loss in both the lumbar vertebrae and femur of OVX mice. Representative microscopic images of the lumbar spine and histological analysis of the trabecular bone volume/total volume ratio in the vertebrae are shown in Figure 3, which is adapted from Tenkumo et al. [75], Scientific Reports, (2020), doi.org/10.1038/s41598-020-65403-4, used under CC BY 4.0. Furthermore, osteoclastogenesis in the lumbar spine was enhanced in OVX mice, and this increase was suppressed by GSE, indicating that GSE may inhibit the OVX-induced rise in osteoclast activity. In OVX rats, the healing of calvarial bone defects was significantly impaired; however, GSE administration restored the healing capacity. Similarly, osseointegration of tibial implants was delayed in OVX rats, but GSE mitigated this impairment, likely by supporting balanced bone remodeling. Regarding the relationship between osteoporosis and dental implants, these findings suggest that oral PACs may support dental implant osseointegration by counteracting the detrimental effects of postmenopausal estrogen deficiency on bone health. A schematic illustration showing how PACs would improve osseointegration of dental implants in patients with PMOP is shown in Figure 4.

## 5. Proposed Mechanisms by Which Proanthocyanidins Enhance Gut Barrier Function via Nrf2 Activation

Oxidative stress is recognized as a major pathogenic factor contributing to the development of inflammatory diseases. Nrf2 is a transcription factor belonging to the basic leucine zipper family. Under physiological conditions, Nrf2 is sequestered in the cytoplasm by Kelch-like ECH-associated protein 1 (Keap1). However, upon exposure to oxidative stress, Nrf2 dissociates from Keap1 and translocates into the nucleus [76]. Nrf2 binds to the antioxidant response element (ARE), thereby activating the transcription of phase II detoxification and antioxidant enzyme genes such as glutamate-cysteine ligase catalytic subunit (GCLC), glutamate-cysteine ligase modifier subunit, heme oxygenase-1 (HO-1), and NAD(P)H quinone dehydrogenase 1 (NQO1), which enhances cellular defense against oxidative stress and provides protective effects [77,78]. Therefore, suppressing Keap1 activates the Keap1/Nrf2 pathway, which helps combat oxidative stress. The Keap1/Nrf2 signaling axis has attracted considerable interest as a key regulator of inflammatory processes, and accumulating evidence supports the involvement of the Keap1/Nrf2 pathway in the development of inflammation-related diseases, as well as its role in suppressing pro-inflammatory signaling pathways [79,80]. Furthermore, some studies have shown that Nrf2-activation can enhance gut barrier function. Urolithins derived from polyphenols, especially urolithin A, along with the synthetic compound 6H-benzo [c]chromene-3,8-diol, reportedly enhanced gut barrier function by activating Nrf2 signaling pathways. Consequently, the expression of tight junction proteins, including claudin 4 and occludin, increased, thereby strengthening the gut barrier [81]. Activation of the aryl hydrocarbon receptor (AHR) by spermidine, a naturally occurring polyamine found in food, reportedly alleviated the intestinal barrier dysfunction associated with colitis [82]. AHR activation triggered the Nrf2 signaling pathway, which enhanced the transcription of antioxidant enzymes such as HO-1, NQO1, superoxide dismutase (SOD)1, and SOD2, and suppressed the phosphorylation of signal transducer and activator of transcription 3 (STAT3), both contributing to anti-inflammatory and barrier-protective effects. Additionally, through AHR-Nrf2 crosstalk, tight junction integrity and intestinal permeability improved via the upregulation of tight junction proteins, including ZO-1, occludin, and claudin-1. Similarly, it was demonstrated that mulberry leaves improved antioxidant capacity and strengthened intestinal barrier function in weaned pigs by activating the Nrf2 signaling pathway and upregulating the activities of antioxidant enzymes [83].

Many studies have shown that PACs exert their health benefits by activating Nrf2 signaling. In in vivo studies using diabetic rats, GSE administration protected the retina and the kidney from hyperglycemic damage, potentially by reducing oxidative stress-induced injury through activation of the Nrf2 pathway [84,85] and alleviated lead-induced oxidative stress in the liver and kidney by activating the Nrf2 signaling pathway [86,87]. Additionally, GSE also prevented the majority of the tissue and molecular damage caused by titanium dioxide nanoparticle exposure [88], and PACs prevented cadmium-induced oxidative damage to the testes [89]. The protective effects of GSE were likely attributable to its potent antioxidant properties, which were associated with the activation of Nrf2 and the upregulation of its downstream genes such as NQO1, HO-1, and GCLC. Regarding oxidative damage in the testis, testicular oxidative damage in a rat varicocele model induced by partial ligation of the left renal vein was attenuated by GSE potentially due to its antioxidant activity and ability to activate the Nrf2 pathway [90]. Recently, a study using T2DM model rats reported that GSE protected against the ferroptosis of pancreatic β cells by activating the Nrf2 signaling pathway [91]. Additionally, although GSE upregulated the expression of Nrf2 and its associated proteins, the protective effect of GSE on ferroptosis was negated when co-transfected with si-Nrf2. This indicated that GSE suppressed ferroptosis by activating the Nrf2 signaling pathway, thereby alleviating β-cell injury and T2DM dysfunction.

**Figure 5:**
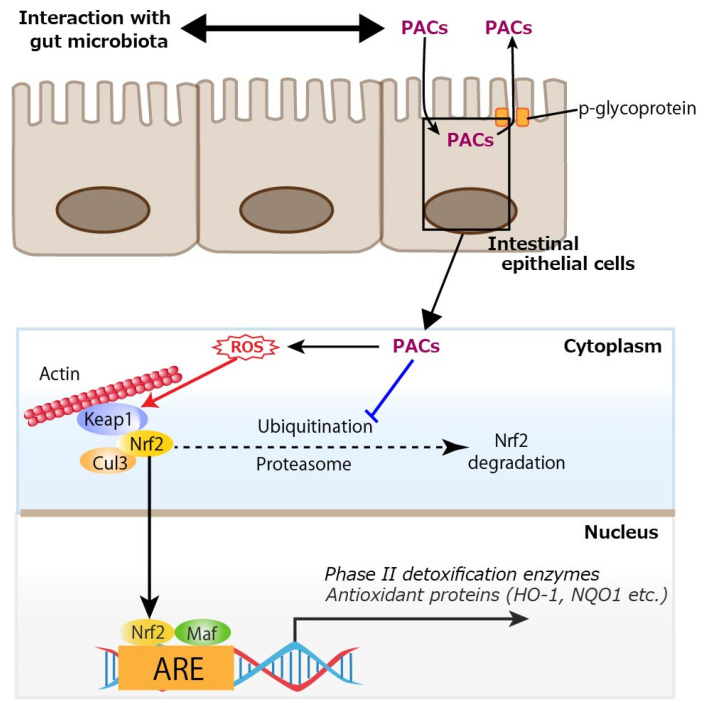
Cellular uptake of proanthocyanidins (PACs) and activation of nuclear factor erythroid 2-related factor 2 (Nrf2) by reactive oxygen species (ROS) produced from PACs. A limited amount of oligomeric PACs (degree of polymerization 2 to 6) could be trans-cellularly incorporated, which would subsequently be excreted via an efflux pump, p-glycoprotein. PACs prevents the ubiquitin-mediated degradation of Nrf2, and ROS generated through the oxidation of PACs activate Nrf2, thereby promoting the transcription of antioxidant enzymes such as heme oxygenase-1 (HO-1) and NAD(P)H quinone dehydrogenase 1 (NQO1). Keap1: Kelch-like ECH-associated protein 1, Cul3: cullin 3, Maf: small Maf proteins, ARE: antioxidant response element. A blue line indicates an inhibitory site, and a red line indicates a site of action.

In addition to the in vivo studies, many in vitro studies have shown that PACs can activate the Nrf2 signaling pathway. Procyanidin C1, a (−)-epicatechin trimer, activated the Nrf2/HO-1 signaling pathway to prevent glutamate-induced apoptosis of HT22 cells, the immortalized mouse hippocampal cell line [92]. An extract purified from lotus seed skin, consisting mainly of dimeric procyanidin B1, procyanidin B2, and procyanidin B4, enhanced the expressions of antioxidant proteins by activating the Nrf2–antioxidant response element pathway in HepG2 cells, a human hepatoblastoma-derived cell line, although it had limited ability to directly scavenge radicals in vitro [93]. Procyanidin B2 also protected MAC-T (mammary alveolar cells-large T antigen) cells from heat stress-induced cell death, oxidative stress, and inflammation, partly by modulating the Nrf2 signaling pathway [94]. Regarding cryobiology, procyanidin B2 attenuated damage to mouse testicular tissue during freezing by inhibiting oxidative stress and apoptosis via activation of the Nrf2/HO-1 antioxidant signaling pathway, leading to increased activity of downstream antioxidant enzymes, and improved mitochondrial kinetic homeostasis [95]. A study using primary rat osteoblasts showed that PACs improved osteogenic activity in osteoblasts impaired by dexamethasone (dex) through the inhibition of oxidative stress, mitochondrial impairment, and apoptosis [96]. Mechanistically, PACs alleviated dex-induced osteoblast damage via activation of the Nrf2 pathway, since Nrf2 knockdown partially reduced the protective actions of PACs. Interestingly, PACs abolished cytotoxicity in rat vascular smooth muscle cells induced by PM2.5, a well-known air pollutant, by activating the Nrf2 signaling pathway with resultant alleviated oxidative stress [97]. Pretreatment with PACs from kiwi leaves (*Actinidia chinensis*) markedly alleviated H_2_O_2_-induced oxidative stress in Caco-2 cells, as evidenced by reduced reactive oxygen species (ROS) production and lower malondialdehyde levels [98]. In the study, the PACs enhanced the activity of antioxidant enzymes (glutathione peroxidase, catalase, total SOD) and upregulated the mRNA expression of key components in the Nrf2 signaling pathway, including Nrf2, HO-1, SOD-1, and catalase in the Caco-2 cells. In MODE-K cells, a murine enterocyte cell line, PACs activated the Nrf2/ARE signaling pathway by preventing the ubiquitin-mediated degradation of Nrf2, thereby enhancing its protein stability and expression, and subsequently modulating essential antioxidant enzymes like HO-1 and NQO1 to trigger cytoprotective responses [99]. Regarding inflammatory cells, PACs alleviated inflammation in LPS-induced RAW264.7 cells, a mouse macrophage cell line, by regulating ferroptosis through the Nrf2/HO-1/Keap1 pathway, suggesting that supplementation with PACs is an effective approach for mitigating inflammation by reducing inflammatory cytokine release and inhibiting ferroptosis [100]. Direct activation of the Nrf2 signaling pathway by PACs requires their uptake into intestinal epithelial cells. In a study of in vitro permeation of oligomeric PACs across a monolayer of Caco-2 cells, a limited amount of oligomeric PACs (degree of polymerization 2 to 6) could be transcellularly incorporated, which could subsequently be excreted via an efflux pump, p-glycoprotein [101]. Similarly, a limited amount of dimeric and trimeric PACs was incorporated into Caco-2 cells [102,103]. To activate the Nrf2 signaling pathway intracellularly, the prooxidative property of PACs would be involved. Polyphenols are known for their antioxidative properties, but their prooxidative potential has also been investigated. A well-known example is tea catechin in the presence of cupric ions, which exhibits prooxidative activity by facilitating DNA cleavage and promoting linoleic acid peroxidation [104]. Specifically, catechin is believed to reduce Cu^2+^ to Cu^+^, and the generated Cu^+^ subsequently induces the formation of ROS, which attacks DNA. Similarly, it has been demonstrated that the anticancer effect of plant polyphenols is driven by intracellular copper mobilization and ROS generation. This prooxidative characteristic of polyphenols is believed to contribute to cancer cell death [105]. Another typical example of the prooxidative action of polyphenols is the antibacterial activity of catechins [106]. This activity is attributed to the production of ROS, such as hydrogen peroxide (H_2_O_2_), generated through the oxidation of catechins as the underlying mechanism. As such, PCAs would also act as prooxidants. Indeed, we previously showed that ROS were produced upon photoirradiation of GSE, which was similar to (+)-catechin; this prooxidant activity was also observed in its bactericidal effect [107]. In addition to interaction with gut microbiota, considering cellular uptake of oligomeric PACs and the prooxidative potential of PACs, ROS produced from trans-cellularly incorporated PACs would activate the Nrf2 signal pathway in intestinal epithelial cells as shown in Figure 5.

## 6. Proposed Mechanisms of Action of Proanthocyanidins

PACs, especially polymeric forms from sources like GSE, help to maintain gut and systemic health through multiple mechanisms.

### 6.1. Strengthening the Gut Barrier

PACs upregulate tight junction proteins (e.g., occludin, ZO-1), which improve intestinal barrier integrity and reduce permeability [9,10,11,12]. This helps prevent harmful substances like lipopolysaccharides (LPS) from entering the bloodstream.

### 6.2. Reducing Inflammation

PACs suppress inflammatory cytokines such as TNF-α and IL-1β [8,12,100], which are involved in systemic inflammation and bone resorption [59,63,74].

### 6.3. Modulating Gut Microbiota

PACs promote the growth of beneficial bacteria—for example, *Akkermansia* (genus) and Lachnospiraceae (family) [12,14]—and reduce harmful ones—for example, Bacteroidota (phylum) and Desulfovibrionaceae (family) [14,15]—further supporting gut health and reducing inflammation.

### 6.4. Activating the Nrf2 Pathway

Under oxidative stress, PACs activate the Nrf2 pathway [84,85,90,91,97,98,99], possibly by causing mild ROS generation after being taken up by intestinal cells [101,102,103,104,105,106,107]. Nrf2 then moves to the nucleus and promotes the expression of antioxidant enzymes like HO-1 and NQO1, enhancing the body’s defense against oxidative stress and inflammation.

### 6.5. Supporting Bone Health

In postmenopausal models, PACs reduce bone loss by suppressing osteoclast activity through inhibition of the RANKL/RANK pathway [74,75], which is activated by inflammatory cytokines. This effect also helps improve dental implant integration in osteoporotic conditions.

**Figure 6:**
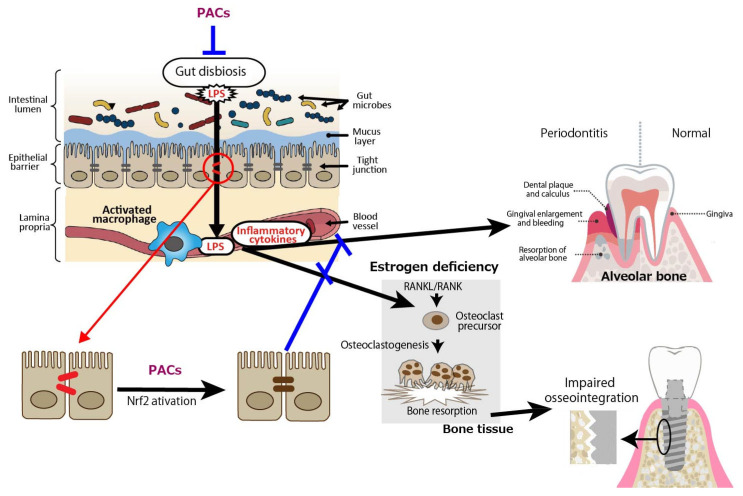
Proposed mechanisms of action of proanthocyanidins (PACs). PACs enhance gut barrier integrity by activating the nuclear factor erythroid 2-related factor 2 (Nrf2) signaling pathway and modulating gut dysbiosis, thereby reducing inflammation of periodontal tissue. These effects also help restore intestinal permeability impaired by estrogen deficiency, ultimately contributing to the prevention of bone fragility causing impaired osseointegration of dental implants. LPS: lipopolysaccharide. Blue lines indicate inhibitory sites, and a red line indicates a site of action.

Overall, PACs act through a combination of antioxidant, anti-inflammatory, microbiota-modulating, and gut-protective effects, largely mediated by Nrf2 signaling and barrier reinforcement, as shown in Figure 6.

## 7. Human Intervention Study of PACs Applied in Dentistry

According to a cross-sectional study that analyzed data from the US National Health and Nutrition Examination Survey 2009–2010 on participants with full-mouth periodontal examination and dietary flavonoids intake data, it was concluded that a higher intake of dietary flavonoids might be beneficial for periodontal health based on periodontal pocket depth (PPD) and clinical attachment level (CAL) as periodontitis markers [108]. PPD is defined as the distance from the gingival margin to the base of the pocket, and CAL is the distance from the cementoenamel junction to the base of the periodontal pocket that indicates the level of attachment of the periodontal tissues to the tooth root. Regarding oral PACs, there have been few reported human intervention studies applied in dentistry so far. One study focused on chronic periodontitis [109] and the other on gingivitis [110]. Detailed information on the two studies is reviewed below.

The first study was a randomized, double-blind, placebo-controlled study, and the objective was to assess the clinical and microbiological outcomes of systemic antibiotic therapy using PACs and secnidazole, a nitroimidazole anti-infective, in patients with chronic periodontitis. A total of 75 individuals diagnosed with chronic periodontitis were randomly assigned into three groups: one treated with secnidazole, another with PACs, and the other with a placebo (25 participants per group). The participants underwent scaling and root planing (SRP) and were given PACs and secnidazole orally for 1 month. Clinical parameters recorded were as follows: plaque index (PI), defined as a quantitative index for evaluating the amount of plaque, the microbial biofilm matrix, on the tooth surface; gingival index (GI), defined as a measure for assessing the health of the gums by visually evaluating qualitative changes in the gingival soft tissues; gingival bleeding index (BI), defined as a periodontal index that assesses the presence or absence of bleeding after gentle probing of the gingival crevice; PPD; and CAL. Findings indicated that both treatment groups exhibited significantly greater reductions in BI, GI, and PPD at both post-treatment and 3-month follow-up compared to the control group. However, there were no notable differences observed in PI and CAL values. Additionally, culturable bacterial counts showed a significant decline following treatment. As such the authors of the study concluded that adjunctive therapy with either PACs or secnidazole alongside SRP in adults with periodontitis is effective in reducing pathogenic bacteria and in improving clinical outcomes to some extent.

The second study aimed to assess the impact of a new nutritional supplement containing oligomeric PACs on tissue response in gingivitis after 21 days of administration. The prospective, randomized, double-anonymized, controlled clinical trial involved 20 participants who were assigned to either an experimental group (PACs intake) or a control group (placebo intake). Each participant attended four clinical visits throughout the 21-day study period, during which the use of any supplementary hygiene methods was not allowed, to evaluate the Silness and Löe gingival index, BI, PI, and gingival brightness. The Silness and Löe gingival index assesses gingival inflammation (color, swelling, and bleeding) on a scale of 0 to 3. The results showed that the control group exhibited a significantly higher Silness and Löe gingival index compared to the experimental group. Consistently, the BI was also lower in the experimental group. Interestingly, the PI revealed 33% more plaque accumulation in the experimental group than in the control group. They concluded that oligomeric PACs appear to positively influence periodontal tissue health even though they failed to reduce plaque accumulation on tooth surfaces.

PACs in the first study were used as a systemic antibiotic therapy agent since it is well-known that they have antibacterial activity [111,112,113,114,115]. However, the bioavailability of PACs is very low [7], such that the reduction of pathogenic bacteria seems to be attributable to the improved periodontal tissue inflammation rather than their antibacterial activity, even though the possibility that residual PACs in the oral cavity may have exerted antibacterial activity cannot be completely ruled out. Indeed, in the latter study, while most of the clinical parameters improved in the PACs group, plaque accumulation worsened, indicating that the antibacterial potential of PACs was not observed. Regarding the PI, no significant effect of the PACs was observed in the first study either. However, both studies suffer from several limitations common to human clinical trials. Most notably, the sample sizes were relatively small—25 participants per group in the first study and 20 in the second—likely resulting in insufficient statistical power. This limitation increases the risk of random error, undermines the reproducibility and reliability of the findings, and limits the external validity and generalizability of the results. Furthermore, the follow-up periods were short—3 months in the first study and 3 weeks in the second—making it difficult to assess long-term outcomes such as relapse prevention or the stabilization of CAL. Given that periodontitis is a chronic disease, a follow-up period of at least 6 months to 1 year or more would be necessary to adequately evaluate sustained clinical effects.

Regarding the topical application of PACs in dentistry, since PACs inhibited biofilm formation of *Streptococcus mutans*, a major dental cariogenic bacterium [116], and *Aggregatibacter actinomycetemcomitans*, a major periodontopathogen [117], adjunctive use of PACs by subgingival application resulted in better clinical outcomes for cases of periodontitis with moderate periodontal pockets [118]. In one study [118], collagen hydrogels containing locally derived PACs were topically applied to evaluate their effects on the nonsurgical treatment of periodontitis, with collagen hydrogels chosen owing to recent research highlighting their potential as scaffolds for periodontal tissue regeneration [119,120]. Accordingly, when delivered with an appropriate carrier, PACs may serve as an effective adjunct to minimally invasive nonsurgical therapy in patients with generalized periodontitis.

## 8. Limitations of the Gut–Oral Axis Hypothesis in Dentistry

The oral cavity hosts the second-largest number of bacteria in the human body, after the gut [121]. Dysbiosis, or an imbalance in microbial communities, in either location can lead to disease. In the oral cavity, dysbiosis is linked to periodontal disease, where deep periodontal pockets—up to 15–20 cm^2^ in severe cases—harbor dense bacterial biofilms [122]. Among these bacteria, the “red complex” (*P. gingivalis*, *Tannerella forsythia*, and *Treponema denticola*) plays a key role in disease progression [123]. Periodontal disease is also associated with systemic conditions such as diabetes, cardiovascular disease, and rheumatoid arthritis, possibly owing to systemic inflammation caused by the translocation of bacterial components like LPS and cytokines into the bloodstream [24,25,26,27,28,29,30,31,124,125,126]. However, evidence remains limited. The gut contains an estimated 600 to 1000 trillion bacteria across >1000 species and is involved in various metabolic and neurological processes [38,39,40,41,42,43]. Emerging research on the “oral–gut axis” suggests that oral dysbiosis can influence the gut microbiota. Animal studies show that oral administration of *P. gingivalis* increases circulating LPS and inflammatory cytokines and disrupts intestinal barrier function [127,128]. In humans with periodontal disease, around 10^9^–10^10^ *P. gingivalis* cells are swallowed daily via saliva [129]. Owing to its relative acid resistance—especially when gastric acidity is reduced—*P. gingivalis* may reach the intestines and influence gut microbiota through the digestive route [130]. Nonetheless, more research is needed to determine the specific gut bacteria affected.

From the perspective of the gut-to-oral connection, there is a report that alveolar bone resorption progressively increases in dextran sulfate sodium (DSS)-induced colitis mice [55]—commonly used as a model for human inflammatory bowel disease—which exhibit local macrophage activation in the colon, impaired intestinal barrier function, and an increase in Gram-negative bacteria [53,54]. It is conceivable that Gram-negative, LPS-producing bacteria increase in the gut, and LPS leaks into the bloodstream through disrupted tight junctions. In addition, inflammatory cytokines produced by activated macrophages may also enter circulation and affect periodontal tissues. This suggests the possibility of a reverse pathway in the gut–oral axis, where changes originating in the gut influence the oral cavity. However, there is still a limited understanding of which oral conditions are influenced by changes in the gut, highlighting the need for further research in this area.

## 9. Conclusions

The possible involvement of PACs in oral health was discussed in this review, with a focus on their effects in the intestine, especially in the colon, where most orally ingested PACs are supposed to reach. It can be expected that PACs ameliorate periodontal diseases as well as improve the osseointegration of dental implants in patients with osteoporosis. Periodontal diseases are exacerbated by LPS leaking into the blood from the site of tight junction disruption and inflammatory cytokines produced from activated macrophages. This mechanism is also observed in osseointegration of dental implants; that is, LPS-induced inflammatory cytokines released from the intestine into the blood are linked to bone loss and subsequent incomplete osseointegration of dental implants. PACs incorporated into intestinal epithelial cells act as a prooxidant and activate the Nrf2 signaling pathway, leading to enhanced gut barrier function. Reduced LPS and inflammatory cytokines in the blood consequently lead to remission of periodontal tissue inflammation, and increased alveolar bone density with enhanced osseointegration of a dental implant. However, the majority of the current evidence provided in this review was derived from in vitro and animal studies, and the available clinical data were limited in both quality and quantity. For instance, both human studies [109,110] employed small sample sizes and relatively short-term treatment, thereby limiting the generalizability of the findings. Thus, potential publication bias and selective reporting may have influenced the overall conclusions.

Although research into oral PACs is ongoing, there are currently only a limited number of human intervention studies demonstrating their efficacy in dentistry. Further studies are required to confirm their effectiveness in humans.
